# The effects of diet and mating system on reproductive (and post‐reproductive) life span in a freshwater snail

**DOI:** 10.1002/ece3.4689

**Published:** 2018-11-16

**Authors:** Josh R. Auld

**Affiliations:** ^1^ Department of Biology West Chester University West Chester Pennsylvania

**Keywords:** aging, caloric restriction, life history, longevity, mating system, phenotypic plasticity, *Physa acuta*, self‐fertilization

## Abstract

The length of the reproductive life span, along with the number/frequency/magnitude of reproductive events, quantifies an individual's potential contribution to the next generation. By examining reproductive life span, and distinguishing it from somatic life span, we gain insight into critical aspects of an individual's potential fitness as well as reproductive and somatic senescence. Additionally, differentiating somatic and reproductive life spans can provide insight into the existence of a post‐reproductive period and factors that shape its duration. Given the known importance of diet and mating system on resource allocation, I reared individual freshwater snails (*Physa acuta*) from 22 full‐sib families under a 2 × 2 factorial design that crossed mate availability (available [outcrossing] or not [selfing]) and diet (Spirulina or lettuce) and quantified aspects of the entire life history enabling me to distinguish reproductive and somatic life spans, determine the total number of reproductive events, and evaluate how the reproductive rate changes with age. Overall, mated snails experienced shorter reproductive and somatic life spans; a diet of Spirulina also shortened both reproductive and somatic life spans. A post‐reproductive period existed in all conditions; its duration was proportional to somatic but not reproductive life span. I evaluate several hypotheses for the existence and duration of the post‐reproductive period, including a novel hypothesis that the post‐reproductive period may result from an increase in reproductive interval with age. I conclude that the post‐reproductive period may be indicative of a randomly timed death occurring as the interval between reproductive events continues to increase. As such, a “post‐reproductive” period can be viewed as a by‐product of a situation where reproductive senescence outpaces somatic senescence.

## 1 INTRODUCTION

The length of the life span, and importantly the *reproductive* life span, plays a critical role in determining individual fitness. Along with the frequency of reproduction and number/size of offspring produced, the reproductive life span is a fundamental life‐history trait encapsulating an individual's potential contribution to the next generation. While life span (and reproductive life span nested therein) varies greatly among species (e.g., Mourocq et al., 2016), we know far less about intraspecific (and intrapopulation) variation in these traits, and understanding variation at this level is critical for an understanding of the potential adaptive evolution of life span. While numerous life‐history studies have focused on events occurring early in the life cycle (e.g., age at first reproduction; Auld, 2010; Escobar et al., 2011), far fewer have evaluated events occurring late in the life cycle (e.g., age at last reproduction; Klepsatel et al., 2013; Curtsinger, 2016); by measuring both we can differentiate the length of the reproductive life span from the somatic life span (Reznick, Bryant, & Holmes, 2006).

The reproductive life span, the timeframe between the first and last reproduction, is subject to resource allocation trade‐offs and senescence (van Noordwijk & de Jong, 1986). While the reproductive life span is necessarily shorter than the somatic life span because of a juvenile/developmental stage (i.e., a pre‐reproductive phase), it may also be shortened when individuals cease to reproduce before they die (i.e., a post‐reproductive phase). This might occur, for example, when the rate of reproductive senescence is faster than the rate of somatic senescence (Croft, Brent, Franks, & Cant, 2015; Kirkwood & Shanley, 2010) or when resource allocation to early‐life reproduction negatively affects the potential for late‐life reproduction (e.g., as predicted by the disposable soma hypothesis; Kirkwood & Shanley, 2005, 2010 ). Under some circumstances, there can also actually be adaptive benefits (e.g., helping kin) that lead to a cessation of reproductive effort (e.g., Pavard, Metcalf, & Heyer, 2008; Cant & Johnstone, 2008). Furthermore, the reproductive rate may vary (e.g., decline) during the reproductive phase and it should not be assumed that a longer reproductive phase necessarily leads to a higher reproductive output. Alternatively, reproductive function might plateau at late ages as seen for mortality in some systems (e.g., *Drosophila*; reviewed in Curtsinger, 2016). As such, we need accurate measures of the duration of the pre‐reproductive, reproductive, and post‐reproductive phases to obtain a complete picture of life span and how it is affected by senescence. Herein, I measure the reproductive life span of a common freshwater snail and relate it to somatic life span, reproductive rate, and reproductive output.

Different patterns of resource allocation between growth/soma and reproduction can be predicted to alter the duration of the somatic/reproductive life span as well as reproductive output. For example, individuals with a larger “budget” of available resources or those following a classic “*r* life‐history strategy” (MacArthur & Wilson, 1967; Pianka, 1970) of maximizing early reproduction would be expected to invest more resources in reproduction early in life relative to individuals with smaller resource budgets or those following alternative life‐history strategies. Note, Stearns (1992) rejected the idea of an *r*‐*K* continuum, and recent work has reframed some of these arguments into a “fast‐slow” continuum (e.g., Salguero‐Gómez et al., 2016). Regardless, of the dichotomy, a larger early‐life investment in reproduction may lead to an overall shorter reproductive life span relative to those that exhibit lower early‐life investment in reproduction (i.e., a “live fast, die young” strategy). While we cannot experimentally “force” individuals to *choose* a different strategy of resource allocation, we can manipulate environmental conditions such that individuals are provided with differing resource “budgets” or made to follow alternative life‐history strategies. For example, by providing individuals a higher quality diet, we can experimentally impose a different energy budget and observe the consequences of this on somatic/reproductive life span and reproductive output. Additionally, we can experimentally alter the life‐history strategy by altering the potential for mating—for example, if individuals are not given access to mating partners, they may be forced to delay the onset of reproduction. Lower early‐life investment in reproduction might alter the pattern of resource allocation and have implications for the duration of somatic/reproductive life span and reproductive output. Furthermore, examining life span and reproductive output under a variety of environment conditions can reveal plasticity that is central to understanding how these traits may be shaped by selection in different environments.

Whenever reproduction stops before death occurs, a post‐reproductive period exists. The existence of a post‐reproductive period has been reported in a variety of species ranging from humans, elephants, and killer whales to guppies, fruit flies, and springtails (Reznick et al., 2006; Tully & Lambert, 2011; Foster et al., 2012; Klepsatel et al., 2013; Lahdenpera, Mar, & Lummaa, 2014; but see Moorad & Walling, 2017). Work by Reznick et al. (2006) has demonstrated that a post‐reproductive period exists in several wild populations of guppies, but the magnitude of the post‐reproductive period did not vary among populations. Nonetheless, a post‐reproductive period is not universal (Croft et al., 2015), so both documenting it and explaining its existence remain important questions in basic research on the expression and evolution of life histories. There are several well‐tested and supported hypotheses that predict the cessation of female reproduction (i.e., menopause) in species with kin interactions when such a shift is beneficial to the female in terms of increasing the survival probability of her offspring and/or grand‐offspring (i.e., the “mother” and “grandmother” hypotheses; Hawkes, O'Connell, Blurton‐Jones, Alvarez, & Charnov, 1998; Shanley & Kirkwood, 2001; Reznick et al., 2006; Pavard et al., 2008; Croft et al., 2015). These hypotheses explain how menopause itself could be beneficial to an individual female because of the indirect benefits to her kin. Nonetheless, they do not explain why such a pattern could be beneficial in species without kin interactions and the observation of a post‐reproductive period in guppies and insects has prompted a number of alternative hypotheses to explain this pattern.

At least two different hypotheses have been previously advanced to explain the existence of a post‐reproductive period in organisms without sophisticated kin interactions. First, Reznick et al. (2006) reported the existence of a post‐reproductive period in guppies that was unrelated to the duration of the reproductive life span. In this well‐characterized system (e.g., Bryant & Reznick, 2004), populations differ in both total life span and reproductive life span, but the post‐reproductive life span appeared to be merely a “random add‐on” at the end of the reproductive period (Reznick et al., 2006; see also Klepsatel et al., 2013 for a similar result in *Drosophila*). Under the “random add‐on” hypothesis, the post‐reproductive period is seen and a purely random period of time after the cessation of reproduction prior to death. Second, Tully and Lambert (2011) proposed an alternative hypothesis suggesting that a post‐reproductive period could actually be beneficial as an “insurance” against haphazardly dying before the completion of the reproductive period. Their model, the “indeterminacy” hypothesis, suggests that a positive relationship between the duration of the post‐reproductive period and variation in the somatic life span could be selected for, and would lead to an adaptive cessation of reproduction even in species with no kin interactions. The central idea of this hypothesis is that a longer post‐reproductive period would be beneficial when there is a lot of variation in somatic life span—if death randomly occurs it would be beneficial to have already completed the reproductive period. They tested their model and found support using springtails (i.e., collembolans; Tully & Lambert, 2011).

The post‐reproductive period might be neither a “random add‐on” nor an “insurance against indeterminacy,” but rather a by‐product of differing rates of reproductive and somatic senescence. If reproductive function senesces more rapidly than survival, reproduction may cease before death, and this could be predicted based on the relative rates of reproductive/somatic senescence and the manner in which reproductive function itself senesces. For example, if an iteroparous individual begins reproducing at a certain rate and their reproductive function senesces, the duration between successive rounds of reproduction (i.e., the reproductive interval) might increase with age. If such an individual died during one of these increasingly long reproductive intervals, it could appear as if a post‐reproductive period existed, but in essence the individual could have reproduced again if it had stayed alive for a longer period of time. This hypothesis would be tested by examining (a) change in reproductive interval across the reproductive life span and (b) the relationship between the reproductive life span and the post‐reproductive life span. If reproductive interval increases with age, and death occurs during an increasingly long reproductive interval, it might appear that the post‐reproductive period is just a random add‐on. Furthermore, if this is the case we would expect no relationship between the reproductive and post‐reproductive life spans. These hypotheses are not necessarily mutually exclusive and support for one may not reject another. In fact, the senescence explanation might make the post‐reproductive period appear to be just a random add‐on, but the senescence hypothesis does not make the same predictions as the indeterminacy hypothesis.

I evaluate support for these hypotheses by manipulating mating opportunities and resources (diet) and tracking the timing of every reproductive event across the life span in a common, simultaneously hermaphroditic, freshwater snail (*Physa acuta*). Previous work (e.g., Auld & Relyea, 2010; Auld, Helker, & Kolpas, 2016) has shown that mating system can affect life span, presumably due to shifts in resource allocation. When individuals have more access to mating partners they initiate reproduction early and their life span in shortened, but the relative duration of reproductive life span is unknown. When individuals are not given access to mating partners, they delay the onset of reproduction (i.e., delayed self‐fertilization; Auld, 2010, Auld & Henkel, 2014), and we can expect a consequent delay in the onset of reproductive senescence. Previous work evaluating the effects of mating system on relative rate of reproductive senescence has shown that reproductive function of mated snails tends to senesce faster than in unmated snails (Auld & Henkel, 2014), but this comparison is only based on the first few reproductive events. Furthermore, diet is known to affect individual condition where individuals fed a higherquality diet exhibit an earlier onset of reproduction and much more rapid reproductive senescence compared to snails reared on a lower‐quality diet (Auld & Henkel, 2014). The implications of these shifts on total reproductive (and post‐reproductive) life span are unknown, but by exploring the effects of mating system and diet (condition) on the duration of life span we can gain insight into the lability of these traits, as might be seen across a range of natural environmental conditions.

## 2 METHODS

Full experimental details are given in Auld and Henkel (2014), where the effects of diet and mating system on age/size at first reproduction (delayed selfing) and early‐life reproductive success (inbreeding depression) are reported. Herein, I report the consequences of these experimental treatments on the age/size at last reproduction and death, namely by examining the distinctions among the somatic, reproductive, and post‐reproductive life spans.

### 2.1 Experimental design

A total of 578 snails from 22 full‐sib families of *P. acuta* were reared individually under a factorial combination of two different diets and two mate‐availability treatments. These snails were the second‐generation descendants of wild‐caught adults from a pond near West Chester, PA. Experimental snails were isolated 3 weeks post‐hatching (well before sexual maturity), and all families were split into two diet treatments—50% were fed a diet of boiled (green‐leaf) lettuce, and 50% were fed a diet of Spirulina flakes (O.S.I.; 41% crude protein [min], 4% crude fat [min], and 6% crude fiber [max]). Both diets were provided ad libitum; Spirulina is considered the higher quality diet. At 5 weeks post‐hatching (just prior to maturity), G_2_ snails in both diet treatments were isolated and split into two mate‐availability treatments—50% remained in isolation throughout their life and 50% were provided with a mating partner for scheduled “conjugal visits.” Mating partners were not related to the experimental snails thereby reducing the chance that mate rejection would occur—this species has been shown to reject closely related mates (Facon, Ravigné, & Goudet, 2006). All mates were marked with a harmless dot of paint (Henry & Jarne, 2007). Mates were added to appropriate experimental containers for 3 hr following each water change and feeding (3 times per week), a time period that is more than sufficient for copulation in both the male and female roles (Auld et al., 2014). Mates were available throughout the remainder of the life span on this same schedule.

For each individual snail, I recorded age at first reproduction (AFR) as well as reproductive success (egg hatching and early juvenile survival). Snails lay their eggs in transparent capsules that are adhered to a substrate, these capsules typically contain a few tens of eggs and are produced every few days following the first reproduction. I collected the first two egg capsules laid by each individual, counted these eggs, and set them aside to quantify hatching success of the G_3_ snails (Auld & Henkel, 2014); adult snails were placed into a new box. Subsequently, I continued to perform water changes, feeding, and mate treatments 3 times per week and each additional egg mass was removed. I recorded the date that each egg mass was produced and continued the experiment until each snail died. As such, the age at last reproduction (ALR) was defined to be the age at which the last egg mass was observed and the age at death (AD) is defined to be the age when the snail died. Therefore, the reproductive life span (RL) for any individual can be defined as ALR‐AFR, and the post‐reproductive life span (PRL) can be defined as AD‐ALR. Note that RL was actually calculated as ALR‐AFR+1, because eggs had to be laid on at least 1 day. The total (somatic) life span (SL) is equal to the age at death. Finally, because the date of each reproductive event (i.e., egg mass deposition) was recorded, I calculated the reproductive interval (RI) as the time between successive reproductions. If more than two eggs masses were produced, RI has multiple values for an individual. These can be related to the age of individuals to evaluate a change in RI across the life span.

### 2.2 Statistical analyses

I analyzed the distribution of somatic life span (SL) and reproductive life span (RL), including the effects of mating and diet, by comparing survival curves. Namely, I compared the survival and reproductive life span of mated/not‐mated snails and lettuce‐fed/Spirulina‐fed snails to assess the effects of mating and diet, respectively. Survival curves were estimated using Kaplan–Meyer tables, and hazard ratios were computed using Cox Proportional Hazards regression analysis and compared using the chi‐squared (*χ* ^2^) test statistic. For each comparison, I calculated age‐specific survival and mortality, and used this to calculate cumulative age‐specific mortality. Because there was no censoring of the data, total cumulative mortality was equal to the number of snails of each type. As in Auld et al. (2016), I calculated *E_j,t_*, the expected number of deaths of type *j* (e.g., where *j* indicates the treatment group, *j = a,b*) on day *t*, using the following formula:

$$E_{j,t}=N_{j,t}*\left(\frac{O_{t}}{N_{t}}\right),$$

where *N_j,t_* is the number of snails of type *j* alive on day *t*,* O_j,t_* is the number of observed deaths of type *j* on day *t*,* N_t_ = N_a,t_ + N_b,t_* is the total number of snails (of both types) on day *t*, and *O_t_ = O_a__,t_* + *O_b,t_* is the total number of observed deaths (of both types) on day *t*. The hazard ratio (HR) is then calculated by taking the ratio of the total number of observed to expected events (over time) in two independent treatments:

$$\mathrm{HR}=\frac{\left(\sum O_{a,t}/\sum E_{a,t}\right)}{\left(\sum O_{b,t}/\sum E_{b,t}\right)}.$$

I also fitted generalized linear mixed models using the *glmer* command in R (v. 2.15.2; *lme4* package v. 0.999999‐0; R Core Team, 2012) to analyze variation in SL and RL. These models included the effects of diet, mate treatment, and their interaction as fixed factors; family was included as a random factor. The interaction between family and diet/mate treatment was included as a random factor to assess among‐family variation in treatment effects (i.e., G × E interactions; Lynch & Walsh, 1998). These models were fit using a Poisson error distribution and used REML when evaluating random effects and ML for fixed effects. The statistical significance of each term was determined using likelihood‐ratio tests comparing models with and without a given term (Bolker, 2008), the difference in log‐likelihoods being chi‐square distributed.

The existence of a post‐reproductive period was assessed using the same methods as Tully and Lambert (2011). In short, I analyzed both a “raw” measure of PRL (AD‐ALR) and a measure standardized by generation time (estimated as family‐mean, treatment‐combination AFR). As in Tully and Lambert (2011), mean AFR is often used to scale life‐history variables (Gaillard et al., 2005). I fitted generalized linear mixed models for these two measures of PRL using the same methods mentioned above. To assess the life span‐indeterminacy hypothesis, I quantified family‐level variation in somatic life span (raw and standardized by RL) as in Tully and Lambert (2011)—an increase in the variance in life span is predicted to be associated with the duration of the post‐reproductive period. To assess the reproductive‐senescence/interval hypothesis, I performed a linear regression of reproductive interval on age—an increase in RI with age could result in the “existence” of a post‐reproductive period. These regressions were done for each treatment combination independently.

## 3 RESULTS

### 3.1 Somatic life span

**Figure 1:**
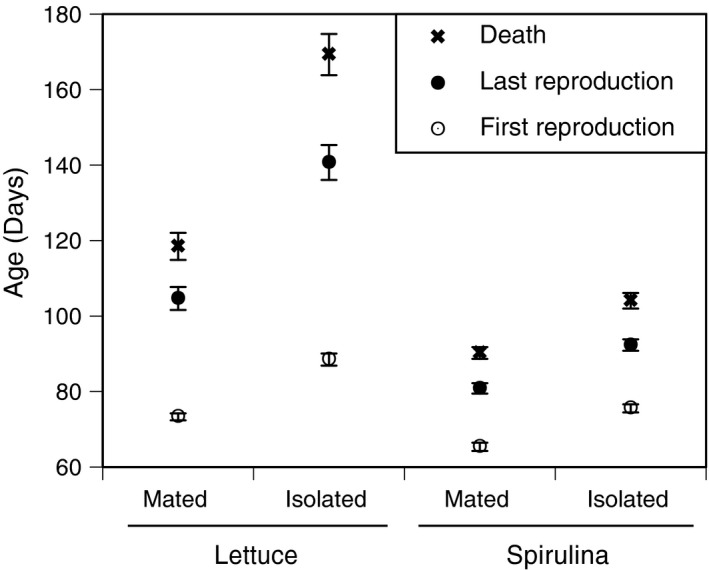
Mean ages at first reproduction, last reproduction, and death (±1 *SE*) for *Physa acuta* reared under two different diets (Lettuce or Spirulina) and two different mate treatments (mated or isolated). Age at first reproduction was reported and discussed in Auld and Henkel (2014) and is redrawn here for comparison. The difference between the age at last reproduction (ALR) and the age at first reproduction (AFR) is the reproductive life span (RL), the difference between age at death at ALR is the post‐reproductive life span (PRL)

**Figure 2:**
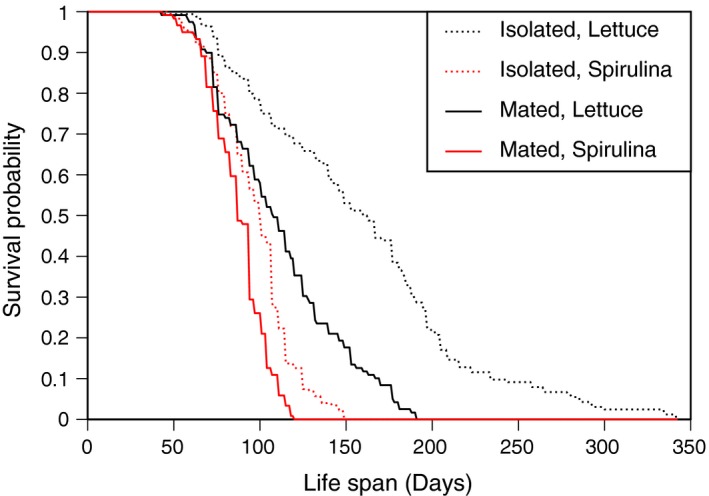
Cumulative survival as a function of age for *Physa acuta* reared on a diet of lettuce (black lines) or Spirulina (red lines). Snails were reared individually and were presented with a mate (solid lines) or remained in isolation (dashed lines)

Consistent with previously observed patterns (Auld et al., 2016), mating reduced somatic life span (Figures 1 and 2, Table 1). Snails that remain in isolation have a longer somatic life span than mated snails (HR* = *1.818, *χ* ^2^ *= *51.5, *p* < 0.001). This effect was observed for snails reared on a diet of lettuce and a diet of Spirulina. Diet also had an effect on life span, where snails fed lettuce lived longer than snails fed Spirulina (HR* = *2.548, *χ* ^2^ *= *135.6, *p* < 0.001). The effect of mating was greater for lettuce‐fed snails compared to Spirulina‐fed snails, as evidenced by the diet*mate interaction (Table 1, Figure 1). The effects of family and both family‐by‐treatment interactions were significant. The majority of the explained variance is attributable to the family‐by‐diet interaction (66.1%) with the family‐by‐mate interaction explaining the remainder (33.9%); the main effect of family explained <0.0001% of the variance. Family‐level means of AFR, ALR, and AD for each treatment combination are plotted in Supporting information Appendix S1: Figure S1.

**Table 1:** Results of likelihood‐ratio tests comparing linear mixed models to assess the effects of diet, mate availability, family, and their two‐way interactions on the somatic life span and the reproductive life span (SL and RL, respectively). Models were constructed using the *glmer* command in R (*lme4* package), see text for further details. The column titled “Estimate (Error)” provides the effect sizes (and *SE*) for fixed effects and explained variance (and *SD*) for random effects. For the Diet effect, “Estimate” shows the effect of lettuce relative to Spirulina; the Mate effect is isolated relative to mated

Trait	Factor	Estimate (error)	$\chi_{df=1}^{2}$	*p*
SL	Diet	0.246 (0.037)	2,690.4	<0.001
	Mate	0.368 (0.037)	1,026.4	<0.001
	Diet*Mate	0.254 (0.167)	229.7	<0.001
	Family	3.14 e^−^^10^ (1.77 e^−^^5^)	445.3	<0.001
	Family*Diet	1.31 e^−^^2^ (1.15 e^−^^1^)	300.3	<0.001
	Family*Mate	6.73 e^−^^3^ (8.20 e^−^^2^)	143.7	<0.001
RL	Diet	0.598 (0.089)	1849.5	<0.001
	Mate	0.517 (0.134)	377.8	<0.001
	Diet*Mate	0.481 (0.051)	85.3	<0.001
	Family	3.46 e^−^^9^ (5.88 e^−^^5^)	600.9	<0.001
	Family*Diet	6.50 e^−^^2^ (2.55 e^−^^1^)	101.6	<0.001
	Family*Mate	1.84 e^−^^1^ (4.29 e^−^^1^)	482.6	<0.001

### 3.2 Reproductive life span

**Figure 3:**
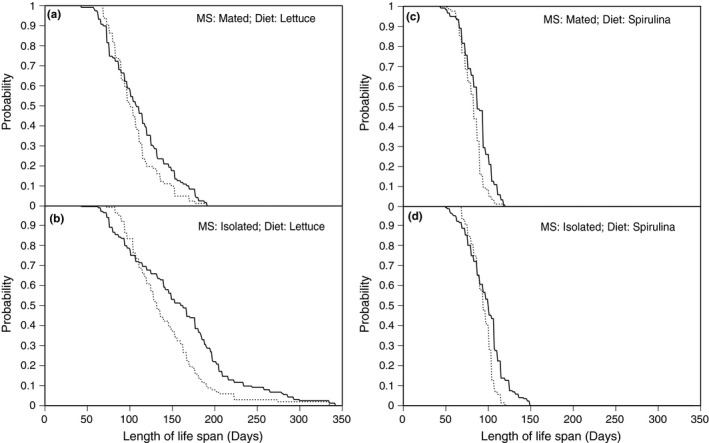
Cumulative survival (solid) and reproductive (dashed) life spans for *Physa acuta* reared under two different diets and two different mating systems (MS). The survival functions are identical to Figure 2, and are redrawn here for comparison with the distribution of reproductive life span (ALR‐AFR)

Of the 578 snails used in the experiment, 336 produced at least one egg mass. As such, information about reproductive (and post‐reproductive) life span are drawn from a smaller dataset than information on total (somatic) life span. Nonetheless, treatment effects on reproductive life span were essentially identical to the pattern observed for somatic life span (Figure 1, Table 1) indicating that a longer somatic life span is correlated with a longer reproductive life span (their relationship is shown in Supporting information Appendix S1: Figure S2). Additionally, the effects of family and both family‐by‐treatment interactions, including the fraction of variance explained, were the same as for somatic life span. This indicates that a fairly random sample of individuals failed to reproduce. I performed a survival analysis, analogous to the comparisons described above, on RL to illustrate the treatment effects on the probability of RLs of various durations. Snails that remain in isolation have, on average, a reproductive life span that is significantly longer than mated snails (HR* = *1.496, *χ* ^2^ *= *13.79, *p* < 0.001). Diet also affected reproductive life span, where snails fed lettuce experienced much longer reproductive life spans than snails fed Spirulina (HR* = *2.329, *χ* ^2^ *= *63.17, *p* < 0.001). The reproductive life span probability distributions are plotted separately for each treatment combination in Figure 3, where they are shown overlapping the survival probabilities.

### 3.3 Post‐reproductive life span

**Figure 4:**
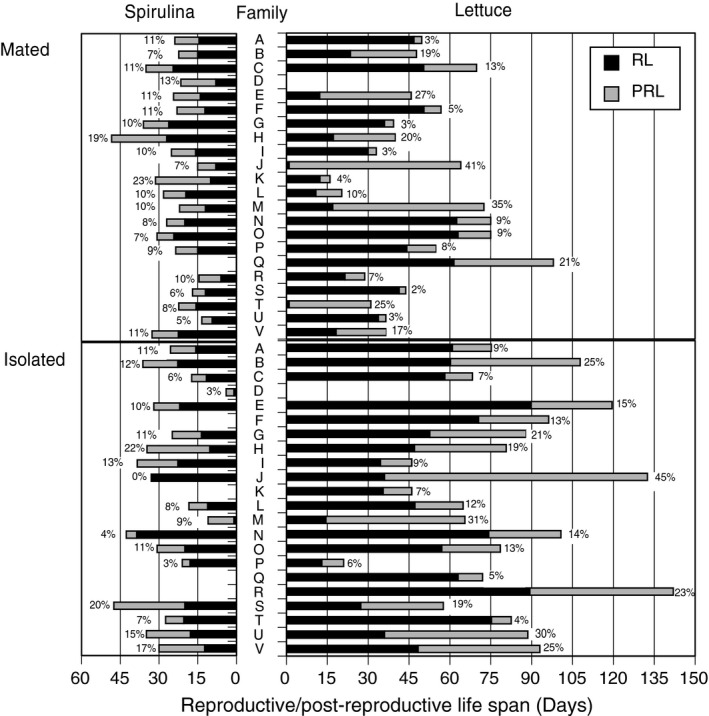
Mean reproductive life span (RL; black bars) and post‐reproductive life span (PRL; gray bars) for 22 full‐sib families of *Physa acuta* reared on one of two different diets (Spirulina or Lettuce) and under 1 of 2 different mate treatments (mated or not [isolated]). The fraction of the total life span that is the post‐reproductive period is indicated as a % for each bar. Missing values indicate that no individual from that family reproduced

**Figure 5:**
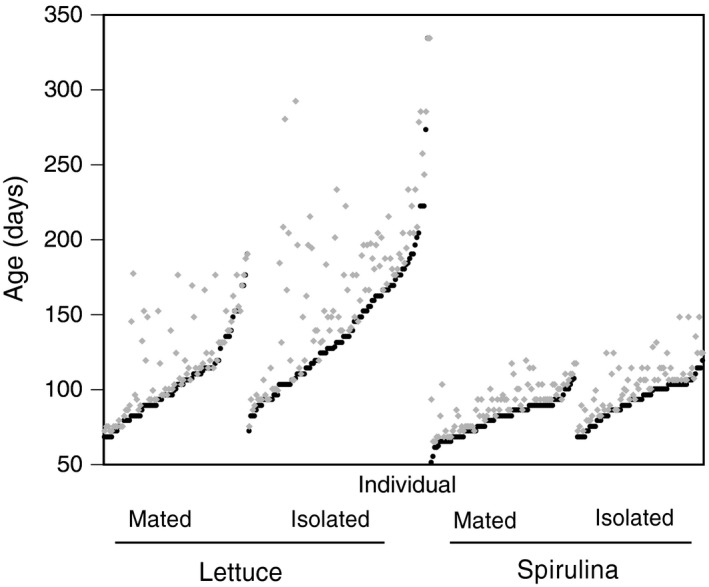
A "life‐history graph" (Carey, Liedo, Muller, Wang, & Vaupel, 1998) showing the relationship between age at last reproduction (ALR) and death (AD). Individuals are plotted, ranked within treatment by increasing ALR (black). Gray symbols represent AD, so the vertical difference between a black and gray symbol is the post‐reproductive life span

A post‐reproductive life span existed under all treatment combinations, and the duration of the PRL was affected by diet and mating (Figure 1, Table 2). The PRL was also significantly affected by family, and by the family‐by‐mate and family‐by‐diet interaction. The main effect of family explained <0.001% of the variance, while the family‐by‐mate interaction accounted for 54.3% of the explained variance and the family‐by‐diet interaction accounted for 45.7%. The PRL (and RL) for each family‐treatment combination is plotted in Figure 4 illustrating that the PRL accounts for 0%–45% of the total life span (grand mean* = *13%). Generally, the PRL was longer for lettuce‐fed snails (mean* = *15.1% of the somatic life span; Table 2) than Spirulina‐fed snails (10.2%), and longer for isolated snails (13.7%) than mated snails (11.9%). However, the effect of mating was much more pronounced for lettuce‐fed snails compared to Spirulina‐fed snails (Figures 1, 4 and 5), similar to the pattern observed for SL and RL. Family variation in PRL is largely accounted for when PRL values are standardized by generation time (mean AFR). The magnitude of PRL is directly proportional to SL (global *r* ^2^ *= *0.36; Supporting information Appendix S1: Figure S3a), but not related to the duration of the RL (Appendix S1: Figure S3b). This would be expected if an individual were to die at a random time during an increasingly long reproductive interval.

**Table 2:** Results of likelihood‐ratio tests comparing linear mixed models to assess the effects of diet, mate availability, family, and their two‐way interactions on the post‐reproductive life span (PRL). PRL was analyzed as both a raw value and after standardization by generation time (PRL/T), where T is family‐level environment‐specific mean AFR. Models were constructed using the *glmer* command in R (*lme4* package), see text for further details. The column titled “Estimate (Error)” provides the effect sizes (and *SE*) for fixed effects and explained variance (and *SD*) for random effects. For the Diet effect, “Estimate” shows the effect of lettuce relative to Spirulina; the Mate effect is isolated relative to mated

Trait	Factor	Estimate (error)	$\chi_{df=1}^{2}$	*p*
PRL	Diet	0.263 (0.171)	593.1	<0.001
	Mate	0.671 (0.177)	422.5	<0.001
	Diet*Mate	0.619 (0.078)	68.0	<0.001
	Family	0 (0)	906.7	<0.001
	Family*Diet	0.269 (0.519)	357.0	<0.001
	Family*Mate	0.321 (0.566)	615.5	<0.001
PRL/T	Diet	0.041 (0.047)	11.86	<0.001
	Mate	0.145 (0.044)	7.70	0.006
	Diet*Mate	0.139 (0.060)	5.25	0.022
	Family	0 (0)	2.51	0.113
	Family*Diet	0.004 (0.062)	1.19	0.275
	Family*Mate	0.003 (0.058)	0.37	0.543

I evaluated the indeterminacy hypothesis by determining the relationship between the PRL and variance in the somatic life span (Table 3). The relationship between PRL and family‐level variance in somatic life span was significant and positive in only one treatment combination (Isolated, Lettuce). This is also the treatment combination with the most variation in both RL and PRL (Figures 4 and 5).

**Table 3:** Results of regression analyses to evaluate whether the post‐reproductive life span (PRL) is associated with variance in life span. Within each treatment combination, family‐mean PRL was regressed on two measures of variance: (a) variance in the somatic life span (varSL), and (b) variance in the somatic life span standardized by reproductive life span (varSL/RL). Statistically significant regression coefficients are in boldface

Treatment (Mate, Diet)	Dependent variable	Regression coefficient (*SE*)	*F*	*df*	*p*
Mated, Lettuce	varSL	0.008 (0.004)	3.85	1, 19	0.065
	varSL/RL	0.002 (0.002)	1.14	1, 16	0.301
Mated, Spirulina	varSL	0.002 (0.007)	0.09	1, 19	0.772
	varSL/RL	0.001 (0.001)	2.37	1, 17	0.142
Isolated, Lettuce	**varSL**	**0.005 (0.002)**	**9.53**	**1, 19**	**0.006**
	varSL/RL	0.001 (0.001)	0.41	1, 19	0.529
Isolated, Spirulina	varSL	0.014 (0.010)	1.86	1, 16	0.192
	varSL/RL	0.001 (0.002)	0.67	1, 12	0.428

**Figure 6:**
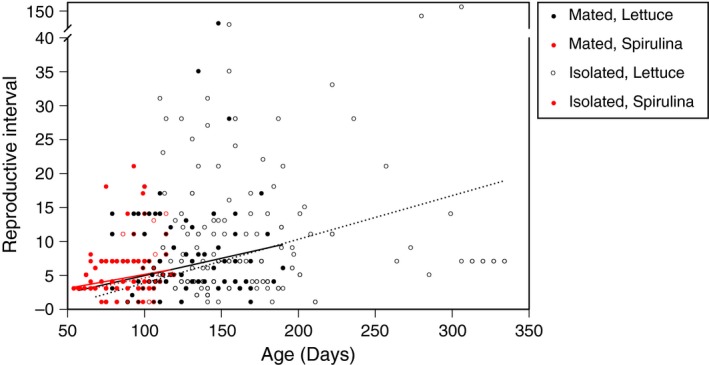
The relationship between reproductive interval (RI, the number of days between successive egg mass depositions) and age for each treatment combination. Linear regressions are shown separately for each treatment combination, with dashed lines coinciding with open circles and solids lines with closed circles. Each regression is significantly positive, see the text for further information. Note the axis break

I evaluated the hypothesis that the PRL is an artifact of a decreasing reproductive rate (i.e., reproductive senescence) by determining how the reproductive interval (RI) changes with age. The RI, the number of days between successive egg mass depositions, increased with age under every treatment combination (Mated, Lettuce: age effect ± *SE = *0.051 ± 0.009, *F* = 35.7, *p* < 0.001; Mated, Spirulina: age effect ± *SE = *0.041 ± 0.011, *F* = 13.7, *p* < 0.001; Isolated, Lettuce: age effect ± *SE* = 0.064 ± 0.007, *F* = 96.3, *p* < 0.001; Isolated, Spirulina: age effect ± *SE* = 0.010 ± 0.009, *F* = 19.7, *p* < 0.001). Across all diet and mating treatment combinations, as individuals age the time between reproductive events increases (Figure 6).

## 4 DISCUSSION

By measuring every reproductive event across the entire life span, I have been able to (a) differentiate the reproductive life span from the somatic life span, (b) document the existence of a post‐reproductive period, and (c) evaluate several hypotheses for the length of these components of the life history.

### 4.1 The duration of the reproductive life span

Reproductive life span varied significantly among the four treatment combinations, and families differed in the degree to which diet and mating system affected the reproductive life span (i.e., G × E interactions). Generally speaking, Spirulina, the higher quality diet, resulted in a shorter reproductive life span, a result which coincides with previous findings that this diet leads to early reproduction and faster reproductive senescence (Auld & Henkel, 2014). This is consistent with research of the effects of diet on life span indicating the caloric restriction tends to elongate life span (Kirkwood & Shanley, 2005; Partridge, Pletcher, & Mair, 2005; Shanley & Kirkwood, 2000; Zajitschek, Hunt, Jennions, Hall, & Brooks, 2008; Zajitschek, Lailvaux, Dessmann, & Brooks, 2012). Additionally, mating with another snail reduced reproductive life span. This is consistent with previous findings in this species (Auld et al., 2016), and may be affected by reduced (early‐life) allocation to both male and female reproductive functions. Reproductive life span may also be shorter for mated snails if compounds transferred along with the sperm (e.g., in the seminal fluid) have negative effects on the recipient, as shown in *Drosophila* (e.g., Chapman, Liddle, Kalb, Wolfner, & Partridge, 1995)—such factors have not been explored in this system, but it is known that manipulative compounds are transferred during copulation in land snails (Lodi & Koene, 2016).

It seems appropriate to consider that treatment effects operate similarly on both the somatic life span and the reproductive life span—both mating and eating a higher quality diet result in a trade‐off where individuals may have higher initial reproductive output (i.e., produce a greater number of eggs) at the expense of a shortened life span. While I did not measure the total number of eggs produced in the present study, previous work (Auld & Relyea, 2010) has shown that net fecundity is directly proportional to life span. Given what we know about inbreeding depression in this species (Auld, 2010; Auld & Henkel, 2014; Escobar, Jarne, Charmantier, & David, 2008; Jarne, Perdieu, Pernot, Delay, & David, 2000), the benefit of outcrossing surely outweighs the survival penalty of mating. In terms of total net reproductive output, it would appear that individuals that consume a lower‐quality diet stand to benefit due to a longer reproductive life span. To the best of my knowledge, no one has examined the degree to which there is variation in the available diet, whether individuals can discern this variation, or whether individuals might express a diet preference. Collectively, the fact that reproductive life span tracks closely with somatic life span is consistent with previous studies (e.g., Reznick et al., 2006; Klepsatel et al., 2013), and is consistent with an interpretation of reproductive function senescing more rapidly than somatic functions.

Toward the end of the reproductive life span, reproduction continued for snails in all treatment combinations, albeit at a decelerating rate. That is, in all combinations of mating system and diet, the reproductive interval increased with age (Figure 6). This yields very little evidence for any sort of late‐life “reproductive plateau” as observed in other species, particularly *Drosophila melanogaster* (e.g., Rauser et al., 2006; Le Bourg & Moreau, 2014; Curtsinger, 2013, 2016 ). This suggests that, at least in this species, there is no need to view older snails as entering a “retired” phase (sensu Curtsinger, 2016), rather it would seem that individuals continue to reproduce, while the reproductive rate is hampered by the process of senescence. Viewed from the resource acquisition and allocation perspective (van Noordwijk & de Jong, 1986), environmental (i.e., treatment) differences in the range of reproductive life span can be easily understood (as discussed in van den Heuvel et al., 2017). Snails that are reared on a higher quality diet can be viewed as having acquired more resources early in life and consequently allocating more resources to early‐life reproduction, with detrimental consequences for the duration of the reproductive life span. Similarly, snails that have access to mates allocate more resources to early‐life reproduction, with a consequent shortening of total reproductive life span. Note that these resources allocated to reproduction are probably attributed to both male and female function (Auld et al., 2014), although male function was not measured here.

### 4.2 The existence of the post‐reproductive life span

A significant post‐reproductive period existed under all treatment combinations, indicating that, regardless of diet or mating system, individuals stop reproducing before they die. This could be viewed, alternatively, as a puzzle to solve or as an artifact of how it was measured. I can say with confidence that, in all treatment combinations, there is a statistically significant difference between the age at last reproduction and the age at death. Therefore, beyond a doubt, a “post‐reproductive” period exists. Nonetheless, I cannot say with confidence that these individuals were in fact “post‐reproductive” (i.e., whether they had “lost the capacity” to reproduce). It is impossible to know whether reproductive function had irreversibly ceased in these individuals or whether they were preparing (albeit more slowly than before) for the production of their next egg mass.

One way to examine the validity of the post‐reproductive period would be to compare its duration to the duration of the final reproductive interval. By taking the difference between an individual's PRL and its final RI, a positive value would indicate that the PRL is longer than the preceding RI. The value of this metric is positive in all treatment combinations (Means ± 1 *SE*); Mated‐Lettuce: 4.94 days (±2.22); Mated‐Spirulina: 4.03 days (±0.97); Isolated‐Lettuce: 21.43 days (±3.64); Isolated‐Spirulina: 7.51 days (±1.46). The grand mean is 10.56 days (±1.40) indicating that the PRL is typically longer than the preceding RI. I still cannot absolutely rule out that snails were capable of reproducing, but these values add strength to the conclusion that a PRL exists.

The observation that the duration of the somatic life span is related to the duration of both the reproductive and post‐reproductive life spans (Supporting information Appendix S1: Figures S2 and S3A) indicates that longer‐lived individuals will experience longer reproductive and longer post‐reproductive periods. Nonetheless, the RL and PRL are not related to each other (Supporting information Appendix S1: Figure S3B), indicating that a longer RL does not equate to a longer PRL. This makes sense when the PRL is viewed as an artifact of a randomly timed death that occurs during an increasingly long reproductive interval. As RI increases late in life, an individual may die shortly after reproducing (a short PRL) or shortly before reproducing (a longer PRL). As such, the relationship between RL and PRL should be non‐existent. This interpretation is also strengthened by the fact that there is a linear relationship between the number of reproductive events and the reproductive life (Supporting information Appendix S1: Figure S4). To some extent, this relationship has to exist, but it also reveals that older individuals continued to reproduce, albeit at a constantly decreasing rate (Figure 6).

### 4.3 The duration of the post‐reproductive life span

The post‐reproductive life span was clearly longer for lettuce‐fed snails than Spirulina‐fed snails. It was also longer for isolated snails compared with mated snails, but this distinction was primarily apparent only for lettuce‐fed snails. Only in one of these four conditions was the length of the PRL related to the variance in SL, as predicted by the indeterminacy hypothesis (Tully & Lambert, 2011). As such, I cannot reject that hypothesis, but it is not well supported by my findings. The random add‐on hypothesis would appear to provide a better explanation—PRL isn't explained by RL. However, given the arguments laid out above, I would contend that the most useful hypothesis is based on reproductive senescence. As these individuals age, their capacity for reproduction decreases and it takes longer and longer to produce their next egg mass. An individual may therefore express a “post‐reproductive” period even if they are capable of producing another egg mass (given enough time).

This interplay between reproductive senescence and somatic senescence deserves more attention, as does the actual mechanism of reproductive senescence. For example, I cannot differentiate the absolute loss of the physiological ability to produce an egg mass from a decrease in this ability that results in an increased reproductive interval. Furthermore, I only investigated female reproductive function, similar changes may also be occurring to male function (Auld et al., 2014), but this experiment did not measure any aspects of male reproductive ability.

## 5 CONCLUSIONS

Reproductive life span is significantly affected by resource allocation and affected by diet and mating. In the wild, where predators and disease abound and pick off weak or slow individuals, the fraction of individuals that are “post‐reproductive” may be minor, but nonetheless investigating these aspects of the life history under lab conditions can yield insight into the interplay between resource allocation and senescence. Generally speaking, individuals senesce because the strength of selection decreases with age (Medawar, 1952; Williams, 1957; Williams, Day, Fetcher, & Rowe, 2006). This means that early‐life reproduction is consistently favored/maximized over late‐life reproduction. The trade‐offs observed here by rearing individuals in different environmental conditions would tend to support the disposable soma view of how senescence occurs (e.g., Kirkwood, 1977; Kirkwood & Rose, 1991; Williams & Day, 2003). Reproducing early in life can accelerate the senescent decline. Future research into the mechanisms of reproductive senescence will be critical to evaluate the reproductive‐interval hypothesis and confirm whether or not the post‐reproductive is really “post‐reproductive” or an artifact of how it is measured.

## CONFLICT OF INTEREST

None declared.

## DATA ACCESSIBILITY

Data from this study are archived in Dryad: https://doi.org/10.5061/dryad.vk5176r.
