# Supplementary material for: The effects of diet and mating system on reproductive (and post‐reproductive) life span in a freshwater snail
Source: Ecol Evol. 2018 Nov 16;8(23):12260–70. doi: 10.1002/ece3.4689 (PMC6303742; doi:10.1002/ece3.4689)
Supplement: Supplementary file 1 [file ECE3-8-12260-s001.docx]

**Appendix Figure 1:** Family means (+ 1 S.E.) for AFR (green), ALR (red), and AD (black) for each treatment combination. When no error bars are shown, data only existed for 1 individual. The x-axis shows the families labeled as letters A-V.

**Appendix Figure 2:** The relationship between reproductive lifespan (ALR-AFR) and somatic lifespan (AD) is shown by plotting family means separately for each treatment combination. Lines show linear regressions; solid lines are for closed symbols while dashes lines are for open symbols.

**Appendix Figure 3:** A) The relationship between post-reproductive lifespan and somatic lifespan is shown by plotting family means separately for each treatment combination. B) A similar plot showing the lack of relationship between post-reproductive lifespan and the reproductive lifespan. The legend is the same for both figures. Lines show linear regressions; solid lines are for closed symbols while dashes lines are for open symbols.

**Appendix Figure 4:** The relationship between the number of reproductions (rounds of egg-mass deposition) and reproductive lifespan (ALR-AFR) is shown by plotting family means separately for each treatment combination. Lines show linear regressions; solid lines are for closed symbols while dashes lines are for open symbols.
